# New Quantitative Method to Identify *NPM1* Mutations in Acute Myeloid Leukaemia

**DOI:** 10.1155/2013/756703

**Published:** 2013-04-09

**Authors:** Sarah Huet, Laurent Jallades, Carole Charlot, Kaddour Chabane, Franck E. Nicolini, Mauricette Michallet, Jean-Pierre Magaud, Sandrine Hayette

**Affiliations:** ^1^Laboratoire d'Hématologie, Centre Hospitalier Lyon Sud, 165 chemin du Grand Revoyet, 69 495 Pierre-Bénite, France; ^2^UMR 5239 CNRS, Faculté de Médecine Lyon Sud, 165 chemin du Petit Revoyet-BP 12, 69921 Oullins Cedex, France; ^3^Service d'Hématologie 1G, Centre Hospitalier Lyon Sud, 165 chemin du Grand Revoyet, 69 495 Pierre-Bénite, France

## Abstract

Somatic mutations in the *NPM1* gene, which encodes for nucleophosmin, have been reported to be the most frequent genetic abnormalities found in acute myeloid leukaemia (AML). Their identification and quantification remain crucial for the patients' residual disease monitoring. We investigated a new method that could represent a novel reliable alternative to sequencing for its identification. This method was based on high-resolution melting analysis in order to detect mutated patients and on an allele-specific oligonucleotide real-time quantitative polymerase chain reaction (ASO-RQ-PCR) for the identification and quantification of the transcripts carrying *NPM1* mutations (*NPM1m*). Few patients carrying known *NPM1m* enabled us to set up a table with the different primers' ΔCT values, identifying a profile for each mutation type. We then analysed a series of 337 AML patients' samples for *NPM1* mutational status characterization and confirmed the ASO-RQ-PCR results by direct sequencing. We identified some mutations in 86 samples, and the results were fully correlated in 100% of the 36 sequenced samples. We also detected other rare *NPM1m* in two samples, that we confirmed by direct sequencing. This highly specific method provides a novel quick, useful, and costless tool, easy to use in routine practice.

## 1. Introduction

Nucleophosmin mutations (*NPM1m*) occur in about one-third of acute myeloid leukaemias (AMLs) [1], and the current classification of myeloid neoplasms defined a recent entity of *NPM1*-mutated AML with distinct biological, clinical, and prognostic features [2]. Moreover, the detection and quantification of *NPM1m* represents a major specific marker for the molecular monitoring of minimal residual disease (MRD) in AML, since it appears as an early initiating event in leukaemogenesis [3, 4]. The expression of this marker is very stable during disease evolution, and the detection of increasing *NPM1m* expression levels seems strongly predictive for impending haematological relapse [5, 6]. Finally, patients' stratification in international clinical protocols and the development of new targeted therapies rely on the *NPM1* status in AML [7]. Thus, the identification of *NPM1m* is of critical importance for the AML patients' admission process. Most of the *NPM1m* identified to date, as the type A mutation (75–80% of cases), are exon 12 frameshift mutations [1, 5, 8] leading to an aberrant accumulation of the protein in the cytoplasm [9]. Several protocols and methods have been developed for the detection of *NPM1m* including DNA sequencing of different mutation-specific RT-PCR assays [10–13], denaturing high-performance liquid chromatography [14], capillary electrophoresis [15], locked nucleic acid-mediated polymerase chain reaction clamping [16], and high-resolution melting analysis [17]. Although these methods possess a high specificity to assess *NPM1 *mutational status at diagnosis, they always require direct sequencing for *NPM1m* characterization, a more expensive and time-consuming method. We therefore investigated a new strategy where (i) mutational status, (ii) distinction between *NPM1* mutation types, and (iii) quantitative value of the identified mutation at diagnosis would be rapidly obtained. 

## 2. Materials and Methods

### 2.1. Samples

A series of 337 AML patients' samples were referred to our laboratory for the initial diagnosis of AML from March 2007 to July 2011. 

### 2.2. DNA and RNA Extraction

Mononuclear cells from bone marrow or blood samples were separated by Ficoll-Hypaque density gradient centrifugation (Histopaque Ficoll-1077, Sigma-Aldrich, Saint Louis, MI, USA) and stored as cellular suspensions containing 10^7^ cells.

We extracted genomic DNA from aliquots of 10^7^ mononuclear cells using the QIAamp DNA Mini Kit and the QIAcube instrument (QIAGEN, Hilden, Germany) according to the manufacturer's instructions, and aliquots containing DNA at 5 ng/*μ*L were prepared.

RNA was extracted using the NucleoSpin RNA II kit (Macherey-Nagel, Düren, Germany), aliquots containing 1 *μ*g RNA were prepared, and reverse transcription was performed as previously described [19]. 

**Figure 1:**
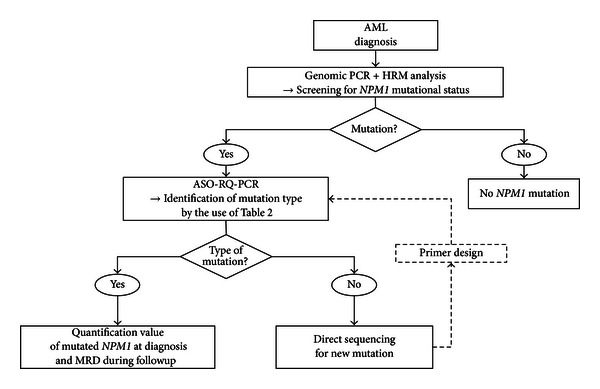
The strategy proposed for identification of *NPM1* mutations. We proceeded in a two-step strategy with a first screening by HRM (high-resolution melting) analysis and then identification and quantification by allele-specific oligonucleotide—(ASO)-RQ-PCR. MRD: monitoring residual disease.

Figure 1 presents an overview of the 2-step strategy we suggest to detect (first step) and identify (second step) the presence of *NPM1m*.

### 2.3. Screening by High-Resolution Melting

First, detection of *NPM1m* was carried out on genomic DNA by PCR and high-resolution melting (HRM) analysis. PCR reactions were performed in a 20 *μ*L final volume containing 5 *μ*L of genomic DNA and 300 nM of each primer (Eurogentec, Seraing, Belgium) (Table 1) [18] with 10 *μ*L of LightCycler 480 Probe Master 2X (Roche Diagnostics, Mannheim, Germany), containing 3.2 mM MgCl_2_ and with 1 *μ*L of ResoLight Dye 20X (Roche Diagnostics) as a nucleotide binding dye. Amplification (defining a 232 bp amplicon) was achieved by 45 cycles of 95°C for 10 secs, 56°C for 15 secs, and 72°C for 15 secs followed by a gene scanning analysis according to the manufacturers instructions by using the LightCycler 480 Real-Time PCR System instrument (Roche Diagnostics). 

### 2.4. ASO-RQ-PCR

When HRM analysis revealed the presence of *NPM1m*, we proceeded to the second step (Figure 1). Identification and quantification of the different mutation types by allele-specific oligonucleotide real-time quantitative polymerase chain reaction (ASO-RQ-PCR) were performed by using 5 distinct RQ-PCR tubes containing a common forward primer, one of five different reverse primers (Eurogentec) designed to specifically target types A, B, C, D, and P mutations [11, 12], and a common probe (Life Technologies Corporation Applied Biosystems, Carlsbad, CA, USA) (Table 1). Each RQ-PCR mixture reaction contained 5 *μ*L cDNA, 300 nM of each primer, 200 nM probe, and 10 *μ*L of LightCycler 480 Probe Master 2X in a total volume of 20 *μ*L. Preheating of the mixture at 95°C for 5 minutes was followed by 45 cycles of a 3-step cycle procedure (10 seconds at 95°C, 40 seconds at 62°C, and 1 second at 72°C). RQ-PCR of the endogenous reference gene *ABL* was accomplished as previously described [19, 20]. All quantitative PCRs were performed using Ipsogen plasmids (Ipsogen Cancer profiler, New Haven, CT, USA), and the assays were found to be linear over at least 5 orders of magnitude (slope: −3.350, −3.480, −3.349, −3.373, −3.305; intercept: 40.27, 40.53, 39.83, 39.66, 39.93 for mutations A, B, C, D, and P, resp.).

### 2.5. Mutational Analysis

Analysis was performed by a comparative cycle threshold (CT) method of relative quantification giving the amount of target, normalized to the *ABL* gene as follows: ΔCT = CT(*NPM1m*) − CT(*ABL*). The mutation type of each sample was identified using Table 2, which indicates the ΔCT profile obtained with each RQ-PCR primer depending on the mutation type.

This table had been previously built using a few patients (1–3 depending on mutation types) carrying known *NPM1m*. The ΔCT values were calculated from each known mutated sample (Table 2). Considering that the smallest CT value (as the ΔCT values) corresponds to the most specific primer, each mutation must be defined by the set of the different primers' ΔCT values obtained:

- mutations A, B, C, and P are clearly identified because the lowest ΔCT values are obtained with primer A, B, C, or P, respectively, as compared to those obtained with the other specific primers;
- in case of mutation D, both primers A and D have low ΔCT value. These two mutations can be discriminated since the ΔCT values of primers B, C, and P are far higher in case of type A than type D.

### 2.6. Sequencing Analysis of *NPM1* Exon 12

To validate our method, we performed direct sequencing on a proportion of positive and negative cases with primer *NPM1*-AS. PCR-amplified fragments from 20 HRM-negative samples and 38 HRM-positive samples (cases with mutation detected by HRM analysis and identified by ASO-RQ-PCR using their ΔCT profile) were sequenced to confirm the results obtained with our strategy. 

## 3. Results and Discussion

**Figure 2:**
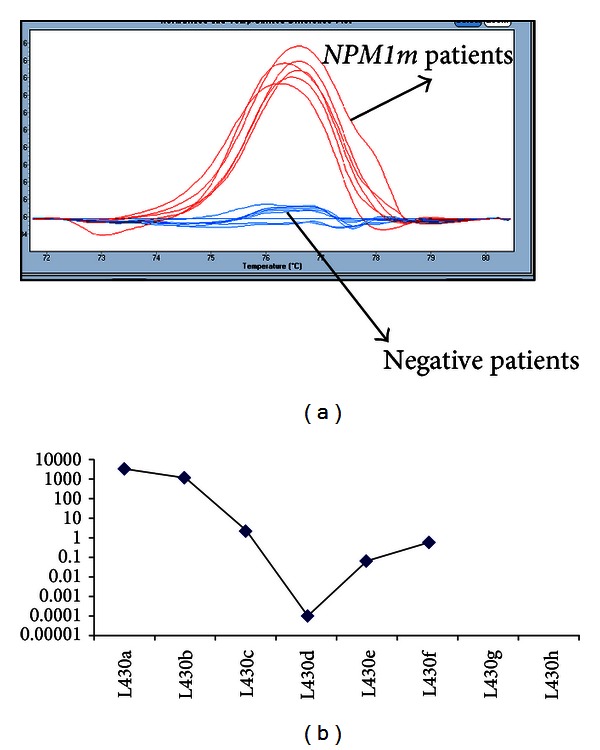
HRM analysis and RQ-PCR of *NPM1* mutations. (a) HRM profiles of 3 patients (in duplicate) harbouring *NPM1* mutations (two A and one B types) compared to 9 negative patients. (b) One example of *NPM1*-A monitoring residual disease by real-time PCR from RNA. The final results were expressed as *NPM1m*/ABL copy number ratios in percent. Analysis was performed on the LC480 Roche device.

Among the 337 samples of AML diagnosis, the HRM screening revealed the presence of *NPM1 *mutation in 88 of them (26.1%). Typical results of HRM analysis are shown in Figure 2(a), allowing distinction between mutated and nonmutated samples. To confirm the absence of mutations and make sure that the new assay does not give false negative results, we investigated by direct sequencing 20 cases that were considered as *NPM1m* negative with the HRM analysis. All the cases proved to be wild-type sequences, which allowed us to consider our strategy as highly specific. 

Among the 88 HRM-positive samples, the ASO-RQ-PCR and the use of Table 2 allowed us to identify the mutation type in 86 samples: 69 carried type A, 10 type B, 1 type C, 5 type D, and 1 type P. The different mutation types obtained using the ΔCT method were confirmed by direct sequencing in 36 samples (30 with mutation type A, 2 type B, 1 type C, 2 type D, and 1 type P), and none of them revealed any other mutation other than the one we identified with our ΔCT method. Thus, the results were fully correlated in 100% of the 36 sequenced samples.

Two samples identified by the HRM screening step showed ΔCT values which did not correspond to any of these mutation profiles showing the following values:

- ΔCT = 0 with primer A and ΔCT = 16 with primers B, C, D, and P for the first sample;
- ΔCT = 15 with primer D and ΔCT = 16 with primers A, B, D, and P for the second one.

We then performed direct sequencing that revealed rare type Q (first sample) and M (second sample) *NPM1* mutations.

The determination of the *NPM1* mutation status in patients with AML is a new urgent requirement for patients enrolled in clinical trials, in order to stratify patients. Although the presence of mutation is currently associated with better outcome, irrespective of the type, its characterization at diagnosis is absolutely necessary for the monitoring of residual disease (MRD) during followup to assess the effectiveness of treatment and may help to identify patients likely to relapse prior to any haematological relapse. For each *NPM1m* patient, the MRD was performed from RNA with a high-sensitivity RQ-PCR method using *NPM1m* specific primers as described above (79 follow-up samples ranging from 0.0014% to 2800%; Figure 2(b) provides an example) and demonstrates that the assays are also suitable for the MRD. 

Although Sanger sequencing represents so far the reference method to identify the mutation types for the first time at diagnosis, this expensive and labor-intensive technique does not represent the ideal way to routinely screen large numbers of patients. Using our strategy, mutation types can be identified since the global CT profiles are unique and surely define exclusively one type of mutation, without requiring sequencing. Besides, this method can be reproduced by each laboratory since it is based on the comparison of ΔCT and not only on raw CT values which could fluctuate between laboratories.

Recently, Barakat et al. [21] described a unique Q-PCR strategy to detect 6 of the most common* NPM1m*. This method presents the advantage to perform only one PCR reaction in a single tube. Nevertheless, it does not allow the identification of the mutation type which must be determined with an additional sequencing step to ensure the MRD. In addition, their assay was designed to screen only the most common *NPM1* mutations and can fail to detect other rare types. Furthermore, among our 88 samples, we detected two rare mutation types (2.3%) that could have been missed or incorrectly identified with a one-tube Q-PCR strategy. Although other mutations are rare, they must not be missed given the importance of *NPM1m *for the molecular followup and therapeutic approaches in clinical trials. We could also avoid the HRM screen step, since samples without *NPM1m *were not amplified by the use of the different specific primers (101 negative remission samples tested as negative control) but, even if the M and Q mutations were amplified by our ASO-RQ-PCR approach, the first screening HRM step avoids missing truly rare but real *NPM1* mutations. We then recommend the use of this method in routine screenings. 

## 4. Conclusions

These results allow us to consider that our strategy is highly specific, and demonstrate in a large group of patients a reliable alternative to *NPM1* sequencing in order to identify the most common *NPM1m*. This method provides a useful and inexpensive tool, easy to use in routine practice, and thus could be included in the genetic diagnosis workup of AML disease.

## Figures and Tables

**Table 1:** Sequences of the different primers and probes.

Gene analysis	Mutations (nucleotides insertion)	Primer	Sequence	Reference
*NPM1* HRM analysis	—	NPM-S (F)	5′ TGGTTCCTTAACCACATTTCTTT 3′	[18]
	—	NPM-AS (R)	5′ GGACAACACATTCTTGGC 3′	—

*NPM1* ASO-RQ-PCR	—	c-NPMl-F (F)	5′ GAAGAATTGCTTCCGGATGACT 3′	[11]
	A (tag)	c-NPM-mut A-R (R)	5′ CTTCCTCCACTGC**CAGA**CAGA 3′	[11]
	B (catg)	c-NPM-mut B-R (R)	5′ TTCCTCCACTGC**CATG**CAG 3′	[11]
	C (cctg)	c-NPM-mut C-R (R)	5′ TTCCTCCACTGC**CACG**CAG 3′	[12]*
	D (cctg)	c-NPM-mut D-R (R)	5′ TTCCTCCACTGC**CAGG**CAG 3′	[12]*
	P (cttg)	c-NPM-mut P-R (R)	5′ TTCCTCCACTGC**CAAG**CA 3′	[12]*
	—	*NPM1* Detection Probe	5′ Fam-ACCAAGAGGCTATTCAA-MGB 3′	[11]

*ABL* ASO-RQ-PCR	—	ENF1003 (F)	5′ TGGAGATAACACTCTAAGCATAACTAAAGGT 3′	[19]
	—	ENR1063 (R)	5′ GATGTAGTTGCTTGGGACCCA 3′	[19]
	—	ENPrl043 detection probe	5′ Fam-CCATTTTTGGTTTGGGCTTCACACCATT-Tamra 3′	[19]

F: forward primer; R: reverse primer; *specific mutation primers were designed based on mutations previously described by Schnittger et al. [12].

**Table 2:** ΔCT obtained for each mutation type with the five different specific primers.

*NPM1* mutation	Specific primer				
	Primer A	Primer B	Primer C	Primer D	Primer P
A	**−3**	14	18	−2	18
B	−2	**−4**	1	−2	3
C	−2	−3	**−4**	−3	3
D	**−3**	1	7	**−3**	5
P	4	15	16	13	**−3**

Negative control (wild type)	No CT obtained				

The ΔCT profiles (i.e., the 5 values obtained in one sample) are specific for the *NPM1m* type.

## References

[1] Falini B, Mecucci C, Tiacci E (2005). Cytoplasmic nucleophosmin in acute myelogenous leukemia with a normal karyotype. *New England Journal of Medicine*.

[2] Arber DA, Brunning RD, Le Beau MM, Swerdlow SH, Campo E, Harris NL (2008). Acute myeloid leukemia with recurrent genetic abnormalities. *WHO Classification of Tumours of Haematopoietic and Lymphoid Tissues*.

[3] Falini B, Martelli MP, Bolli N (2011). Acute myeloid leukemia with mutated nucleophosmin (NPM1): is it a distinct entity?. *Blood*.

[4] Vassiliou GS, Cooper JL, Rad R (2011). Mutant nucleophosmin and cooperating pathways drive leukemia initiation and progression in mice. *Nature Genetics*.

[5] Schnittger S, Kern W, Tschulik C (2009). Minimal residual disease levels assessed by NPM1 mutation-specific RQ-PCR provide important prognostic information in AML. *Blood*.

[6] Kristensen T, Møller MB, Friis L, Bergmann OJ, Preiss B (2011). NPM1 mutation is a stable marker for minimal residual disease monitoring in acute myeloid leukaemia patients with increased sensitivity compared to WT1 expression. *European Journal of Haematology*.

[7] Falini B, Gionfriddo I, Cecchetti F, Ballanti S, Pettirossi V, Martelli MP (2011). Acute myeloid leukemia with mutated nucleophosmin (NPM1): any hope for a targeted therapy?. *Blood Reviews*.

[8] Rau R, Brown P (2009). Nucleophosmin (NPM1) mutations in adult and childhood acute myeloid leukaemia: towards definition of a new leukaemia entity. *Hematological Oncology*.

[9] Falini B, Nicoletti I, Martelli MF, Mecucci C (2007). Acute myeloid leukemia carrying cytoplasmic/mutated nucleophosmin (NPMc+ AML): biologic and clinical features. *Blood*.

[10] Ottone T, Ammatuna E, Lavorgna S (2008). An allele-specific RT-PCR assay to detect type A mutation of the nucleophosmin-1 gene in acute myeloid leukemia. *Journal of Molecular Diagnostics*.

[11] Gorello P, Cazzaniga G, Alberti F (2006). Quantitative assessment of minimal residual disease in acute myeloid leukemia carrying nucleophosmin (NPM1) gene mutations. *Leukemia*.

[12] Schnittger S, Schoch C, Kern W (2005). Nucleophosmin gene mutations are predictors of favorable prognosis in acute myelogenous leukemia with a normal karyotype. *Blood*.

[13] Dvorakova D, Racil Z, Jeziskova I (2010). Monitoring of minimal residual disease in acute myeloid leukemia with frequent and rare patient-specific NPM1 mutations. *American Journal of Hematology*.

[14] Ammatuna E, Noguera NI, Zangrilli D (2005). Rapid detection of nucleophosmin (NPM1) mutations in acute myeloid leukemia by denaturing HPLC. *Clinical Chemistry*.

[15] Noguera NI, Ammatuna E, Zangrilli D (2005). Simultaneous detection of NPM1 and FLT3-ITD mutations by capillary electrophoresis in acute myeloid leukemia. *Leukemia*.

[16] Thiede C, Creutzig E, Illmer T (2006). Rapid and sensitive typing of NPM1 mutations using LNA-mediated PCR clamping. *Leukemia*.

[17] Tan AY, Westerman DA, Carney DA, Seymour JF, Juneja S, Dobrovic A (2008). Detection of NPM1 exon 12 mutations and FLT3—internal tandem duplications by high resolution melting analysis in normal karyotype acute myeloid leukemia. *Journal of Hematology & Oncology*.

[18] Boissel N, Renneville A, Biggio V (2005). Prevalence, clinical profile, and prognosis of NPM mutations in AML with normal karyotype. *Blood*.

[19] Gabert J, Beillard E, van der Velden VHJ (2003). Standardization and quality control studies of “real time” quantitative reverse transcriptase polymerase chain reaction of fusion gene transcripts for residual disease detection in leukemia—a Europe Against Cancer Program. *Leukemia*.

[20] Beillard E, Pallisgaard N, van der Velden VHJ (2003). Evaluation of candidate control genes for diagnosis and residual disease detection in leukemic patients using “real-time” quantitative reverse-transcriptase polymerase chain reaction (RQ-PCR)—a Europe against cancer program. *Leukemia*.

[21] Barakat FH, Luthra R, Yin CC (2011). Detection of nucleophosmin 1 mutations by quantitative real-time polymerase chain reaction versus capillary electrophoresis: a comparative study. *Archives of Pathology and Laboratory Medicine*.

